# A non-canonical binding interface in the crystal structure of HIV-1 gp120 core in complex with CD4

**DOI:** 10.1038/srep46733

**Published:** 2017-04-21

**Authors:** Liang-Wei Duan, Hui Zhang, Meng-Ting Zhao, Ji-Xue Sun, Wen-Li Chen, Jian-Ping Lin, Xin-Qi Liu

**Affiliations:** 1 State Key Laboratory of Medicinal Chemical Biology, College of Life Sciences, Nankai University, Tianjin 300071, China; 2 Research Center for Immunology, School of Laboratory Medicine, Henan Collaborative Innovation Center of Molecular Diagnosis and Laboratory Medicine, Xinxiang Medical University, Xinxiang 453000, China; 3 State Key Laboratory of Medicinal Chemical Biology, College of Pharmacy, Nankai University, Tianjin 300071, China

## Abstract

Numerous crystal structures of HIV gp120 have been reported, alone or with receptor CD4 and cognate antibodies; however, no sole gp120/CD4 complex without stabilization by an antibody is available. Here, we report a crystal structure of the gp120/CD4 complex without the aid of an antibody from HIV-1 CRF07_BC, a strain circulating in China. Interestingly, in addition to the canonical binding surface, a second interacting interface was identified. A mutagenesis study on critical residues revealed that the stability of this interface is important for the efficiency of Env-mediated membrane fusion. Furthermore, we found that a broad neutralizing antibody, ibalizumab, which targets CD4 in the absence of gp120, occupies the same binding surface as the second interface identified here on gp120. Therefore, we identified the possibility of the involvement of a second gp120-CD4 interaction interface during viral entry, and also provided a reasonable explanation for the broad activity of neutralizing antibody ibalizumab.

Initially synthesized as a heavily glycosylated gp160 precursor^1^, the human immunodeficiency virus type I (HIV-1) envelope glycoprotein (Env) is cleaved enzymatically by cellular proteases before binding to the cell surface as two non-covalently associated subunits: gp120 and gp41. Gp120 is the receptor binding subunit^2^ that interacts subsequently with the primary receptor CD4 and a co-receptor (either CCR5 or CXCR4) and undergoes a series of entry-related conformational changes. Gp41 is responsible for the fusion of the viral and cellular membranes^3^ by rearranging from a four-helix collar consisting of discontinuous helices containing N- and C-terminal heptad repeats to a thermodynamically stable six-helix bundle structure^4^,^5^,^6^,^7^.

In addition to its critical role in the life cycle of HIV-1, Env is also the sole target on the surface of HIV-1 virions for antibody-mediated neutralization^4^,^8^. Thus, the structure of HIV-1 Env has been studied extensively. Since Kwong and colleagues reported the first crystal structure of a monomeric HIV-1 gp120 core nineteen years ago^9^, a wide variety of structures of gp120 and its outer domain have been determined using soluble two-domain CD4 and co-receptor mimics, as well as with numerous antibodies that target the CD4 binding site (CD4bs) or the outer domain of gp120^10^,^11^,^12^,^13^. Furthermore, some crystal structures of the unliganded gp120 core have been determined successfully, although it seems that the antibody alone or in combination with CD4 is essential for crystallization of gp120, by acting as stabilizing agents and crystallizing chaperones^14^. Recently, structures of pre-fusion full-length gp120 from an engineered, cleaved HIV-1 Env trimer, termed BG505 SOSIP.664 gp140, and a native, cleaved HIV-1 Env trimer referred to as EnvΔCT, have been resolved at relatively high resolution^15^,^16^. Furthermore, a gp140 trimer with a liganded CD4 complex has also been reported^17^. However, to the best of our knowledge, the structure of a binary complex of HIV-1 gp120 and soluble CD4 has not been reported.

HIV-1 CRF07_BC is a recombinant form that mainly circulates in Northwest China^18^. To define precisely the conformational change of HIV-1 CRF07_BC gp120 induced only by the engagement of soluble CD4, ruling out an influence of co-receptor mimics, we solved the crystal structure of the HIV-1 gp120 core with an extended stem of variable loop 3 (V3)^19^,^20^, which is designated as core_V3e_, in complex with the N-terminal two domains (D1D2) of CD4^9^,^21^ (hereafter referred to as CD4_D1D2_). Compared with the crystal structure of pre-fusion gp120 in the soluble BG505 SOSIP.664 gp140 trimer, our CD4-bound gp120 core_V3e_ structure showed certain small differences^22^. Unexpectedly, a novel non-canonical binding interface between HIV-1 gp120 and CD4 was found, for the first time, based on crystal packing. Residual mutagenesis on this interface was harmful to Env-mediated cell-cell fusion and pseudotyped HIV-1 infection, indicating that this interface is important for HIV-1 fusion and entry. Moreover, based on the structural similarity with the ibalizumab-CD4 complex, the non-canonical interface may provide clues for the broad and potent antiviral activity of ibalizumab^23^.

## Results

### Overall structure of CRF07_BC gp120 core_V3e_ and CD4_D1D2_

**Figure 1:**
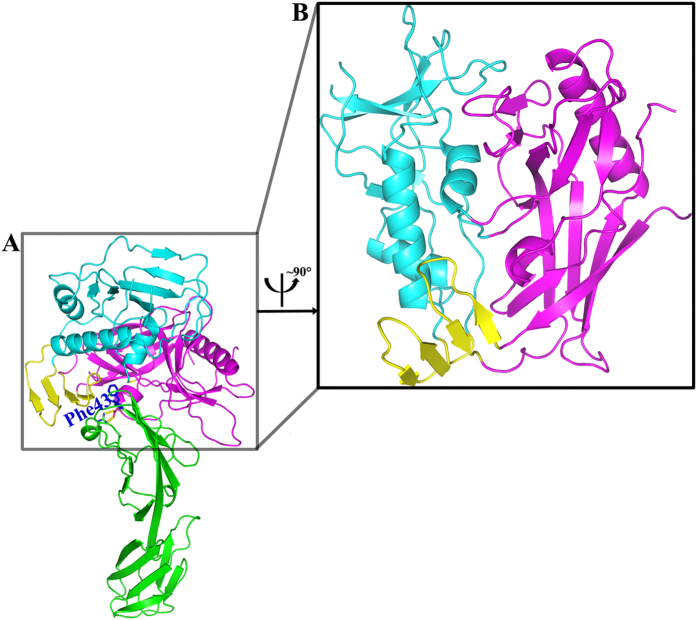
Overall structure of CRF07_BC gp120 core_V3e_ and CD4_D1D2_ complex. (**A**) The complex of gp120 core_V3e_/CD4_D1D2_. Phe43 from CD4, the critical residue in maintaining the overall structure of CRF07_BC gp120 core_V3e_ and CD4_D1D2_ complex, was shown as blue sticks. The inner domain, outer domain, and bridging sheet of gp120 were presented as cyan, magenta, and yellow ribbons, respectively. CD4_D1D2_ is shown in green ribbon. (**B**) Side view of the gp120 core_V3e_, colored as in (**A**).

We attempted to obtain crystals of the binary complex comprising HIV-1 gp120 and D1D2 of human CD4 using a series of gp120 crystallization variants, which contained different truncations of the first three variable loops (V1–V3), and deletions of the N and C termini. However, only the gp120 variant containing 10 additional residues in the stem of the V3 loop could produce crystals of the complex. Monoclinic crystals of the gp120 core with an extended V3 stem (core_V3e_) from HIV-1 CRF07_BC in complex with two-domain CD4 (CD4_D1D2_) were obtained successfully and used for data collection. The complex structure was solved at 2.47 Å (Table S1; Fig. 1A).

The overall structure of this binary complex is similar to previously reported counterparts in CD4-containing ternary structures. As expected, the gp120 core contains three domains: the outer domain, the inner domain, and the bridging sheet (Fig. 1A,B). CD4 D1 is bound into a pocket formed at the nexus of three gp120 domains, in which Phe43 plays a critical role in the interaction between gp120 and CD4^9^ (Fig. 1A). Likewise, the Nη1 and Nη2 atoms of CD4 R59 form double hydrogen bonds with the Oδ1 and Oδ2 atoms of HIV-1 gp120 D368 (numbering based on HXBc2 gp160). Overall, the interactions observed in this complex were conserved compared with those reported previously.

### The non-canonical interacting interface between HIV-1 gp120 and CD4 in the binary complex

**Figure 2:**
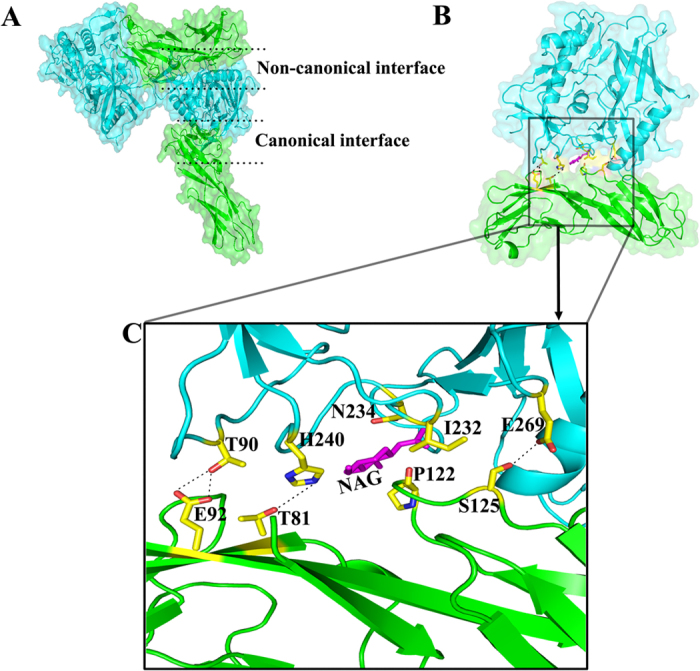
Non-canonical interface in the CRF07_BC gp120 core_V3e_ and CD4_D1D2_ complex. (**A**) Full view of CRF07_BC gp120 core_V3e_ and CD4_D1D2_ complex from two neighboring asymmetric units. Gp120 and CD4 were shown in cyan and green ribbon respectively, and both were superimposed on space-filling models. The area between the upper two dashed lines and the area between the lower two dashed lines represented the non-canonical interface and canonical interface, respectively. (**B**) Full view of the non-canonical interface between gp120 core_V3e_ and CD4_D1D2_ in the alternative asymmetric unit. (**C**) Zooming-in of the non-canonical interface. Side chains of the residues involved in hydrogen bonds that stabilize the non-canonical interface were shown as sticks colored according to atomic types: yellow for carbon, blue for nitrogen and red for oxygen. NAG was shown in magenta. The involved hydrogen bonds were presented as dashed lines. Gp120 was shown in cyan and CD4 was shown in green in all of these pictures. Abbreviations: E, Glu; T, Thr; H, His; N, Asn; P, Pro; I, Ile; S, Ser; NAG, N-acetyl glucosamine.

When we solved the crystal structure of the complex in the asymmetric unit, a distinct gp120/CD4 binding pattern in which gp120 engages with both domains of CD4_D1D2_ emerged unexpectedly (Fig. 2A). A second binding interface between HIV-1 gp120 and CD4, comprising residues T90, N234-linked N-acetylglucosamine (NAG), H240, and E269 from gp120; and residues T81, E92, P122, and S125 from CD4, was identified (Fig. 2B,C). We named it as the non-canonical binding interface. Specifically, within the interface, the side chain of gp120 T90 forms double hydrogen bonds with the carboxylate group of CD4 E92. Additionally, the side chain of H240 makes a hydrogen bond with the hydroxyl group of T81, and the Oε2 atom of E269 forms a hydrogen bond with the side chain of S125. Furthermore, the N234-linked NAG packs closely with P122. By contrast, I232 of gp120 might contribute a repulsive force locally because of its hydrophobic nature (Fig. 2C). The NAG linked to N234 is located in a crevice between gp120 and CD4, where there is enough space to allow for a full glycan chain (Fig. S1). Compared with the canonical binding surface, interactions at the second binding interface comprise primarily hydrogen bonds and are therefore much weaker. This second interface buries a surface area of 471 Å^2^, which is much smaller than the 940 Å^2^ in the primary gp120-CD4 interface (Table S2). As the canonical interface is also present in this complex structure, the second binding interface is probably a result of crystal packing. To verify the physiological relevance of the second binding interface, we examined whether the interface is involved in HIV-1 mediated membrane fusion by performing a mutagenesis study of the involved residues.

### The second binding interface is important for HIV-1 CRF07_BC mediated membrane fusion and viral entry

**Figure 3:**
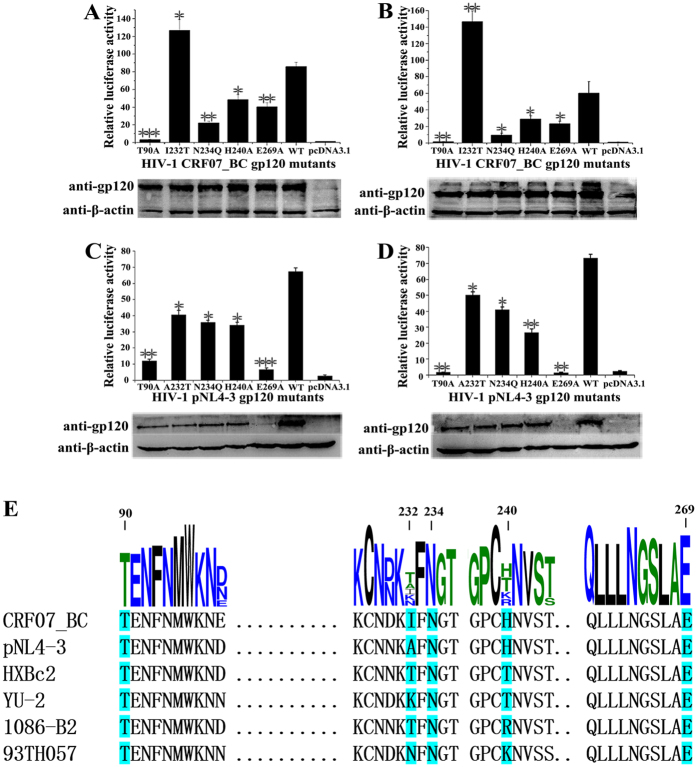
The non-canonical interface was involved in the membrane fusion and cell entry of HIV-1. (**A**–**D**) Relative luciferase activities of different gp120 mutants from HIV-1 strains CRF07_BC (A,B) and pNL4-3 (**C**,**D**). The full-length gels used for cropping were shown in Fig. S4. (**A**,**C**) The relative luciferase activities of different gp120 mutants T90A, I232T/A232T, N234Q, H240A and E269A were determined by Env-mediated cell-cell fusion assay. (**B**,**D**) The relative luciferase activities of different gp120 mutants T90A, I232T/A232T, N234Q, H240A and E269A were determined by cell-associated pseudotyped infection assay. Results are presented as mean ± SEM. Student’s t-test, *P < 0.05, **P < 0.01, ***P < 0.001, compared with the WT group. Three independent experiments were performed for statistics. (**E**) Alignment of gp120 sequences of CRF07_BC and pNL4-3 with strains from various subtypes of HIV-1. The size of each amino acid was drawn to its weight of conservation at the particular location. The residues used for mutations in this study were shaded cyan and numbered on top. Subtypes of HIV-1 strains used in the alignment: HXBc2, subtype B; YU-2, subtype B; 1086-B2, subtype C; 93TH057, subtype A/E.

To address the potential biological importance of the second binding interface, we introduced single point mutations on the gp120 subunit of HIV-1 CRF07_BC Env. These mutations included T90A, H240A, and E269A, which could disrupt hydrogen bonds on the interface; N234Q, which could abolish the formation of NAG; and I232T, which theoretically could introduce a hydrogen bond with the main-chain O atom of CD4 Gly123 via the side chain of threonine. The effects of these mutations were first investigated using a luciferase reporter-based Env-mediated cell-cell fusion assay^24^, in which the cell-cell fusogenic ability is proportional to the observed luciferase activity. All of these mutations that disrupt the interaction at the second binding interface reduced the cell-cell membrane fusion ability, especially the T90A mutant, which disrupts double hydrogen bonds: T90A almost completely abolished cell-cell fusion (Fig. 3A). All these Env proteins were expressed at similar levels in Cos-1 cells according to a western blotting assay. By contrast, the hydrogen bond-introducing I232T mutant increased the fusion ability (Fig. 3A). The membrane fusion activity of the Env mutants was further verified by a cell-associated Env-pseudotyped HIV-1 entry assay. As shown in Fig. 3B, the result agreed well with that of the cell-cell fusion assay. Thus, these results suggested that the second binding interface is important for Env-mediated cell-cell fusion and viral entry of HIV-1 CRF07_BC.

HIV-1 CRF07_BC is a recombinant form derived from subtype B and C; however, the vast majority of its Env region is derived from subtype C, which accounts for almost half of HIV-1 infections worldwide. To extend the potential role of this second binding interface to other HIV-1 subtypes or circulating recombinant forms (CRFs), we further verified the corresponding single point mutations on gp120 in a widely used pNL4-3 strain, a subtype B virus, using both the Env-mediated cell-cell fusion assay and Env-pseudotyped HIV-1 entry assay. The results of hydrogen bond-disrupting mutants in both assays were in good agreement with those in CRF07_BC, except for E269A, which gave uncertain results because of its low expression level (Fig. 3C,D). It should be pointed out that, compared with the wild- type, in pNL4-3, the hydrogen bond-introducing A232T mutant exhibited a decrease in the ability to support cell-cell and virus-cell fusion, a phenomenon that requires further investigation. Most of the tested residues along the non-canonical interface are conserved in different virus strains (Fig. 3E).

**Figure 4:**
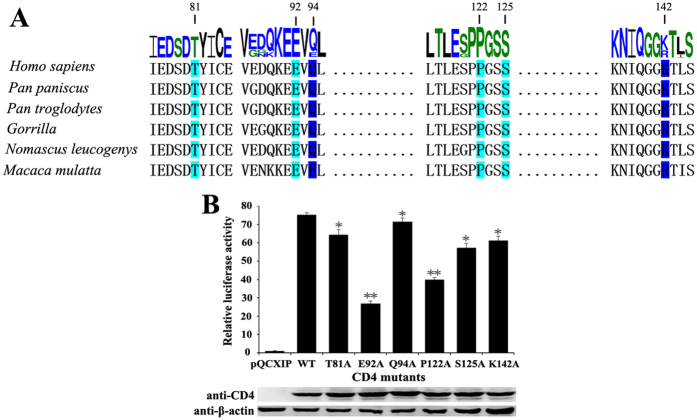
CD4 residues involved in the non-canonical interface affected the membrane fusion and cell entry of HIV-1. (**A**) Alignment of CD4 sequences of *Homo sapiens* with different species. The size of each amino acid was drawn to its weight of conservation at the particular location. The conserved residues and non-conserved residues used for mutations in this study were numbered on top and shaded in cyan and blue, respectively. (**B**) The luciferase activities of CD4 mutants T81A, E92A, Q94A, P122A, S125A and K142A were determined by CD4-associated pseudotyped infection assay. Results were presented as mean ± SEM. Student’s t-test, *P < 0.05, **P < 0.01, compared with the WT group. Three independent experiments were performed for statistics. The full-length gels used for cropping were shown in Fig. S4.

To verify these observations further, we also generated mutations on the CD4 side along the non-canonical interface. HeLa cells overexpressing CD4 and CCR5 were used as the receptor cells for HIV-1 pseudoviral infection. The sequence of this region in CD4 is conserved in various primate mammals (Fig. 4A). Among six CD4 mutants, T81A and S125A abolished one hydrogen bond between the two molecules and reduced the efficiency of infection. E92A abolished two hydrogen bonds and reduced infection more efficiently. P122A also reduced viral infection significantly, which might have resulted from destabilization of the local conformation and interference with the interaction of glycan chain attached to N234 of gp120. Two other mutants, Q94A and K142A, which are variable residues in CD4 of primate animals and are proximal to this interface, were chosen to test the specificity of this interface in HIV-1 infection. As expected, these two mutants had less effect on HIV infection than other mutants (Fig. 4B).

Collectively, our data revealed that the second binding interface between HIV-1 gp120 and CD4 plays a potentially important role in the process of HIV-1 fusion and entry.

### Molecular dynamics simulation of the gp120/CD4 complex stabilized by the non-canonical interface

A 100 ns molecular dynamics (MD) simulation was performed to study the stability of the non-canonical interface of the gp120-CD4 complex. As the harmonic constraint was applied, the root mean square deviation (RMSD) was steady at 0.9 Å during the first 5 ns of the trajectory. It increased quickly to 6 Å with the constraint released, before decreasing and stabilizing at 3.5 Å. At the beginning of the MD simulation, the residues of gp120 and CD4 on the non-canonical interface were not oriented well to form hydrogen bonds. However, after a 20 ns MD simulation, with the orientation of the residues induced and fitted with each other, stable hydrogen bonds were formed. Further extensive simulation indicated that the non-canonical interface could be stable.

The occupancy of the residues on the non-canonical interface during the last 10 ns of the trajectory was calculated. Residues N88, T90, H240, and Q354 in gp120 and K1, K2, E92, Q94, and E119 in the CD4 contributed to critical hydrogen bonds, thereby stabilizing the non-canonical interface of the complex. In addition, the binding free energy between gp120 and CD4, as calculated by the MMPBSA (molecular mechanics Poisson-Boltzmann surface area) method was −18.99 kcal/mol, indicating that the combination of gp120 and CD4 via the non-canonical interface could be a spontaneous process. The structure of the simulated dimer based on the non-canonical interface was similar to that observed in the crystal structure (Fig. S2).

### Comparison of HIV-1 gp120 core_V3e_ with previously determined gp120 structures in different conformations

**Figure 5:**
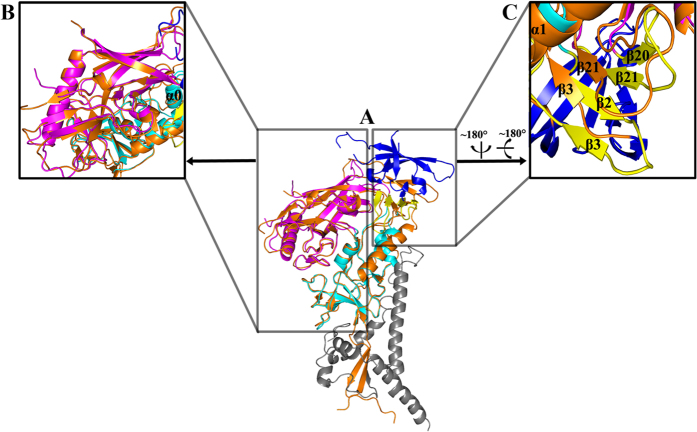
Comparison of CRF07_BC gp120 core_V3e_ with pre-fusion engineered BG505 SOSIP.664 gp140 (PDB code 5CEZ). (**A**) CRF07_BC gp120 core_V3e_ was superimposed to pre-fusion engineered BG505 SOSIP.664 gp140. CRF07_BC gp120 core_V3e_ was colored as in Fig. 1A. Different regions of BG505 SOSIP.664 gp140, i.e., V1/V2, gp120 core and gp41, were shown in blue, orange, and gray, respectively. (**B**) Zooming-in of V4 region between CRF07_BC gp120 core_V3e_ and BG505 SOSIP.664 gp140. (**C**) Zooming-in of V2/V3 between CRF07_BC gp120 core_V3e_ and BG505 SOSIP.664 gp140. The corresponding β-strands were labeled.

Next, we superimposed the gp120 core_V3e_ onto the gp120 component of the pre-fusion engineered BG505 SOSIP.664 gp140 crystal structure at 3.0 Å resolution (PDB code 5CEZ), which is the highest resolution structure in the pre-fusion state published to date^25^. Generally, gp120 in the two structures was similar (Fig. 5A); however, there were discernible differences in the V4 loop and bridging sheet region (Fig. 5B,C). Variation in the V4 loop is associated with HIV evasion from the host immune system^26^,^27^,^28^. Therefore, this difference might reflect the adaptation of various viral strains (Fig. 5B). In the gp120 core_V3e_ structure, strand β2 (residues 119–122) following the long α1 helix (residues 99–117) and strand β20 (residues 423–425) engage in forming the bridging sheet; thus, together with strand β3 (residues 200–202) and β21 (residues 432–434), a stable four-stranded bridging sheet is formed (Fig. 5C). By contrast, in the pre-fusion gp120 structure, the strand β2 region extends from the long α1 helix to form V1/V2 loop, which is consistent with a previous observation by Julien *et al*.^15^. At the same time, strand β20 also exists as an extended loop (Fig. 5C). As a result, the four-stranded bridging sheet does not form in the pre-fusion gp120. Furthermore, both strands β3 and β21 of the gp120 core_V3e_ in the bridging sheet have an obvious shift compared with their positions in the pre-fusion gp120 structure, leading to the formation of parallel sheets and hence the stabilization of the local conformation (Fig. 5C). In addition, a loop (residues 65–73) in pre-fusion gp120, which is also observed in other gp120 structures in the CD4-bound conformation, forms a regular helix (α0) in the current gp120 core_V3e_ structure (Fig. 5B).

**Figure 6:**
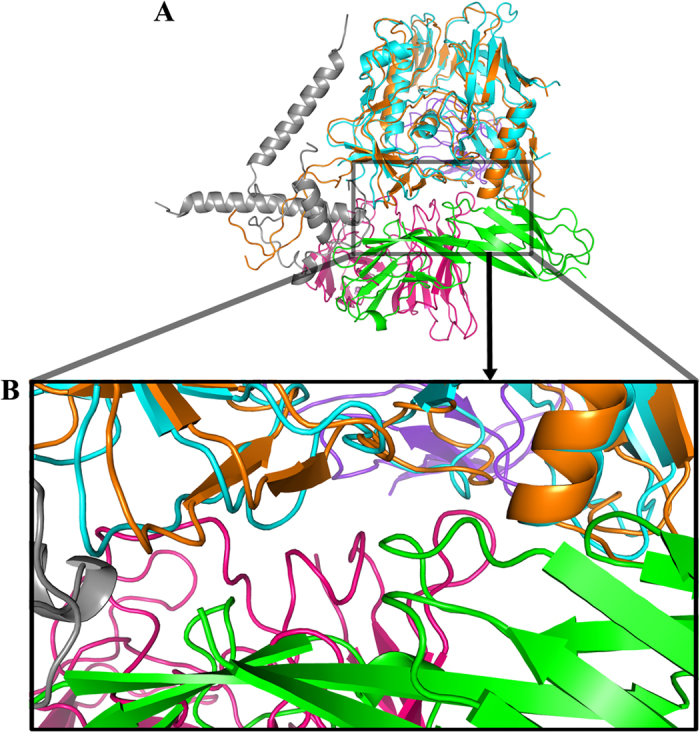
The non-canonical interface in the CRF07_BC gp120 core_V3e_-CD4_D1D2_ complex overlaps with the interface between gp120 and 8ANC195 in BG505 SOSIP.664 Env-sCD4-17b-8ANC195 complex (PDB code 5THR). (**A**) Full view of two complexes, only one gp120 was shown for simplicity. Two complexes were superimposed based on gp120 region. The interfaces in two complexes were marked in a rectangle. Gp120 and CD4 of CRF07_BC gp120 core_V3e_-CD4_D1D2_ complex were shown in cyan and green respectively. Gp120, gp41 and CD4 of BG505 SOSIP.664 Env-sCD4-17b-8ANC195 complex were shown in orange, gray and purpleblue respectively. (**B**) Zooming-in of the interfaces in two complexes.

Recently, the structure of the gp140 trimer complexed with bound CD4, which is stabilized by antibody 17b and 8ANC195, has also been reported (PDB code 5THR)^17^. The gp120 core_V3e_ is also quite similar to gp120 in the CD4-bound gp140 trimer (Fig. 6A). Interestingly, the position of antibody 8ANC195 in CD4-liganded gp140 complex overlaps partially with CD4 in gp120-CD4 dimer formed on the non-canonical interface (Fig. 6B). Furthermore, the local conformation of residues in gp120 along the non-canonical interface is conserved in these structures (Fig. S3). 8ANC195 can interfere with viral entry by interacting with both gp120 and gp41, suggesting that the non-canonical interface might be involved in the conformational change of the envelope protein during viral entry.

### The neutralization activity of ibalizumab might rely on the second binding interface

Ibalizumab is a humanized IgG4 monoclonal antibody of murine origin that binds to the interface between CD4 D1 and D2 and blocks the entry of HIV-1 potently^23^. The ibalizumab binding site on CD4 does not overlap with the binding site of major histocompatibility complex (MHC) class II molecules; therefore, this antibody is safe and well tolerated when infused into patients. Owing to its exceptional breadth and potency, as well as its non-immunosuppressive capability, ibalizumab is currently being evaluated as a potential anti-HIV-1 therapeutic agent in a phase III clinical trial^29^,^30^. Despite significant research effort, the precise mechanism of the inhibition of HIV-1 entry by ibalizumab remains poorly understood.

**Figure 7:**
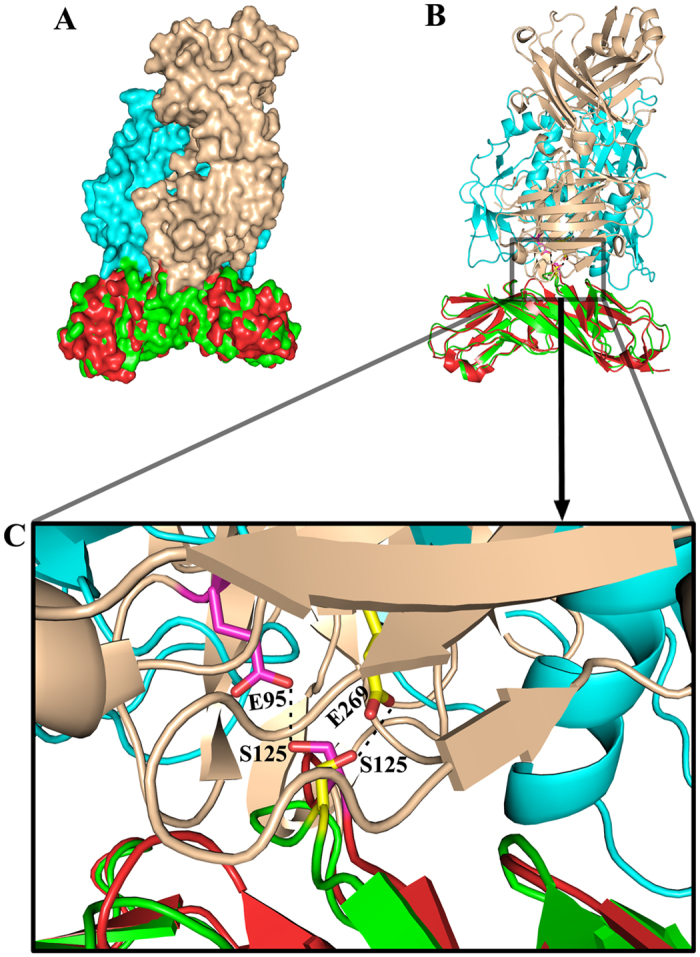
The non-canonical interface in the CRF07_BC gp120 core_V3e_-CD4_D1D2_ complex overlaps with the interface of ibalizumab-CD4 complex (PDB code 3O2D). (**A**) Full view of two complexes shown in space-filling model. Two complexes were superimposed based on CD4 region. CRF07_BC gp120 core_V3e_-CD4_D1D2_ complex were colored in cyan and green for gp120 and CD4 respectively; ibalizumab-CD4 complex were colored in wheat and red for ibalizumab and CD4 respectively. (**B**) Two complexes were shown in ribbon and colored as in (**A**). The non-canonical interface in CRF07_BC gp120 core_V3e_-CD4_D1D2_ complex and the interface in ibalizumab-CD4 complex were marked in a rectangle. (**C**) Zooming-in of the interface. The residues involved in conserved interactions in two complexes were shown in stick model and colored in yellow (gp120-CD4) and magenta (ibalizumab-CD4) for carbon atoms respectively. The dashed lines indicate the comparable hydrogen bonds existing in two complexes. Abbreviations: S, Ser; E, Glu.

A crystallographic study of ibalizumab in complex with CD4 D1D2 suggested that the epitope of ibalizumab is located at the surface and also involved D1 and D2 of CD4; therefore, we tried to characterize the mechanism of ibalizumab by comparing the structure of the ibalizumab-CD4 complex (PDB code 3O2D) with the gp120-CD4 complex formed through the second binding interface. Superimposition of the CD4 molecules from the two complexes revealed that the ibalizumab epitope is positioned on the same region of CD4 as that of the non-canonical binding interface engaged with HIV-1 gp120 (Fig. 7A). Notably, in the ibalizumab-CD4 complex, S125 of CD4 also forms a hydrogen bond with the Oε2 atom of E95 on the heavy (H) chain of ibalizumab, which might recapitulate the interaction between CD4 S125 and gp120 E269 (Fig. 7B,C). Consequently, the binding sites on CD4 for gp120 and ibalizumab are located in close proximity to each other and are mutually exclusive. On the basis of this finding, we speculate that HIV-1 gp120 cannot continue to bind CD4 through the second interface when CD4 is engaged by ibalizumab. This is consistent with the observation that both the bivalent IgG and monovalent Fab forms of ibalizumab could effectively block viral entry with nearly equal inhibition, at least for certain HIV-1 isolates^23^. Therefore, the second binding interface between HIV-1 gp120 and CD4 might be critical for the activity of ibalizumab.

## Discussion

In this study, we solved the crystal structure of a binary complex of HIV-1 gp120 and CD4, thus contributed to our understanding of the conformational rearrangements that HIV-1 gp120 undergoes during the course of virus infection. As described previously, engagement of HIV-1 gp120 by CD4 leads to the formation of a four-stranded bridging sheet, which disrupts the stabilization of the five-stranded β-barrel structure of V1V2 at the apex of Env trimers^9^. The V1V2 β-barrel then disassembles into loops and moves to the periphery of trimeric Env^31^,^32^. At the same time, the core of gp120 also moves sideways as a whole, with almost no conformational change. Next, the V3 loop, largely underneath V1V2, is exposed and protrudes its co-receptor binding tip toward the target cell membrane, rendering gp120 poised for co-receptor binding^10^,^33^,^34^. Finally, co-receptor binding probably drives gp120 to completely dissociate from gp41^35^. Thereby, gp41 could be transformed into a six-helix bundle and provide sufficient energy to drive the fusion of the viral and host cell membranes^4^.

**Figure 8:**
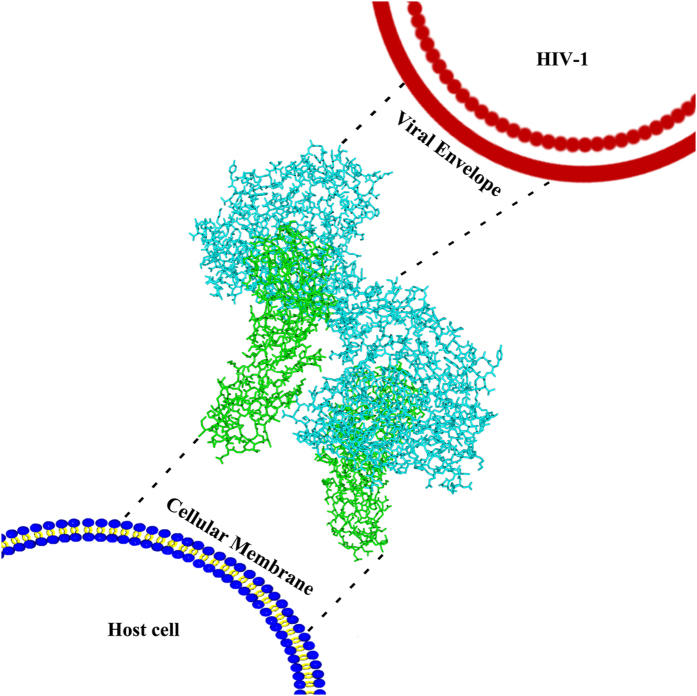
Schematic representation of the membrane fusion between HIV-1 and host cell. The gp120 and CD4 were shown as cyan and green sticks, respectively. Dashed lines indicated the proposed direction from proteins to corresponding membranes.

Surprisingly, in our crystal structure, besides the canonical gp120-CD4 interaction site, gp120 could also contact CD4 through a novel, non-canonical interface. We could not preclude the possibility that this second interface is derived solely from crystal packing; however, our mutagenesis data suggested that this interface is important for the fusion activity of gp120. Intriguingly, the CD4 site engaged by gp120 through this new interface is recognized well by ibalizumab, an antibody with broad anti-HIV activity. The high efficacy of ibalizumab has long been a puzzle because of its special targeting epitope, a site located on CD4 rather than on gp120 or gp41, which are targeted by nearly all other neutralizing antibodies^13^,^36^,^37^,^38^. If our speculation holds true, then it will provide a reasonable explanation for the activity of ibalizumab, i.e., the site recognized by ibalizumab on CD4 is a critical site for the full engagement of gp120 with CD4 during viral infection. Usually there are only 14–20 spikes on every mature viral envelope, so they are dispersed with large distances between them^39^. However, when the viral and host cell membranes fuse, at least 2–3 spikes are needed to work in concert at the contact site to accomplish fusion^40^. During this process, the gp120 in the first spike might engage CD4 at the canonical site initially, followed by the gp120 from neighboring spike engaging the same CD4 at the second binding interface. As a result, the conformational change induced by CD4 engagement will be amplified, and two spikes will be coordinated to initiate the subsequent fusion process. In our crystal structure, two gp120s could engage the same CD4 at two individual sites from the same direction. Additively, two CD4 engaged the same gp120 also point to the same direction, opposite to gp120. Therefore, their spatial arrangement is in agreement with their relative positions during the attachment of viral and cell membranes (Fig. 8).

In summary, our crystal structure of gp120-CD4 complex reveals a new possibility for the involvement of a second gp120-CD4 interaction interface during viral entry, and also provides a reasonable explanation for the broad activity of the neutralizing antibody ibalizumab. Further experiments are needed to validate this speculation; however, our observations provide an interesting avenue that might lead to a more complete understanding of the HIV-1 mediated membrane fusion process between the virus and the host cell.

## Materials and Methods

### Plasmids and molecular cloning

A series of constructs containing different truncations of the first three variable loops (V1–V3) was amplified by overlap polymerase chain reaction (PCR) using the gene encoding HIV-1 CRF07_BC gp145, which was a generous gift from Dr. Yiming Shao (Chinese Center for Disease Control and Prevention, Beijing, China), and cloned into the pMT/BiP/TEV-His expression vector, which was modified from the pMT/BiP/V5-His plasmid (Invitrogen, USA) by replacing the region including the V5 epitope with the tobacco etch virus (TEV) recognition site to allow the specific removal of the C-terminal 6×His tag of the fusion proteins. For construct of HIV-1 CRF07_BC gp120 coreV3e, residues 121 to 203 in the V1/V2 loop were replaced by the tripeptide Gly-Ala-Gly (GAG) and residues 302 to 323 in the V3 loop were replaced by the hexapeptide Gly-Gly-Ser-Gly-Ser-Gly (GGSGSG). The full-length Env proteins were cloned into a mammalian expression plasmid, pSRH NLA/S/Av (kindly provided by Dr. Eric Hunter, Emory University, GA, USA), to generate pSRHS-gp160. The point mutants of full-length Env were generated using the Quick Change Site-Directed Mutagenesis kit (Strategene, La Jolla, CA) according to the manufacturer’s instructions. The fragment encoding the N-terminal two domains (D1D2) of human CD4 (residues 1–181) was amplified by PCR and cloned into the pMT/BiP/TEV-His expression vector as above. All constructs were confirmed by sequencing.

### Cell culture and transfection

Drosophila Schneider S2 cells were cultured in SFX-Insect medium (Hyclone, USA) at 27 °C without CO_2_ and were split at the density of ~6 × 10^6^ cells/ml every 3 days. 293 T, HeLa, TZM-bl and COS-1 cells were maintained in Dulbecco’s modified Eagle’s medium (DMEM) supplemented with 10% FBS (fetal bovine serum), penicillin (100 U/ml) and streptomycin (100 μg/ml), in a humidified atmosphere containing 5% CO_2_ at 37 °C. Drosophila S2 cells and the mammalian cells were transfected using reagents Cellfectin II and Lipofectamine 2000 (Invitrogen, USA) respectively following the manufacturer’s recommendations.

### Establishment of stable cell lines of S2

The pMT/BiP/TEV-His harboring gp120 or CD4 along with the pCoBlast selection vector encoding Blasticidin S deaminase was used to obtain stably transfected S2 cells (ratio19:1). Stable protein expressing cell lines were selected by addition of 25 μg/ml Blasticidin S (Invitrogen, USA) to the SFX-insect culture medium after transfection for 48 h. The selection medium was replaced every 3 days and positive clones were established in ~3 weeks.

### Expression and purification

Stably transfected S2 cells were grown in shaker flasks to a density of 8×10^6^ cells/mL and were induced with 0.5 mM CuSO_4_ for protein expression. 3–5 days after induction, S2 cell suspension was centrifuged for 15 min at 4000 rpm to remove the cells, and the supernatant was concentrated by ultrafiltration using a 10 kDa cutoff membrane at 4 °C. Subsequently, binding buffer (50 mM Tris, pH 8.0, 500 mM NaCl) was added to the concentrated supernatant to adjust the pH of the medium. Protein was purified by affinity chromatography from the supernatant using a Ni-NTA column (Qiagen, Germany) followed by size exclusion chromatography (SEC) using a HiLoad 16/60 Superdex 200 column (GE Healthcare, Sweden) pre-equilibrated with the binding buffer. Protein fractions were pooled, concentrated, and quantified using adsorption at UV 280 nm.

### Binary complex preparation

Purified gp120 was incubated with CD4 D1D2 at 1:1.2 ratio of molecular mole for over 30 min at room temperature. The complex was further purified by a HiLoad 16/60 Superdex200 size exclusion column in 10 mM HEPES, pH 7.5, and 350 mM NaCl. Purified complexes were concentrated to ~40 mg/mL and used for initial crystal screening. For crystal optimization, the complex was digested with TEV protease at pH 8.0, and further subjected to treatment with Endo F1 and Endo F3 at pH 5.5 for deglycosylation, both steps lasting 12 h at 20 °C. The deglycosylated complex was then purified using a HiLoad 16/60 Superdex 200 column equilibrated 10 mM HEPES, pH 7.5, and 350 mM NaCl. Purified deglycosylated complexes were concentrated to ~10 mg/mL for crystal optimization.

### Crystallization, data collection and structure determination

Crystallization trials were carried out by the sitting-drop vapor-diffusion method at 293 K. Drops were formed by mixing equal volumes of the complex and reservoir solution from commercially available preformulated screening kits (Hampton Research, USA). Primary crystals with weak diffraction were obtained using untreated complex. To produce crystals suitable for structural analysis, the complex was subjected to digestion by TEV protease as well as Endo F1 and Endo F3. The monoclinic high quality crystals were produced in the crystallization condition containing 0.1 M HEPES sodium, pH 7.4, 11% PEG 4000 and 10% 2-propanol. Crystals were soaked in mother liquor supplemented with 20% glycerol for 1 min and flash frozen in liquid nitrogen.

X-ray diffraction data were collected at 100 K using a wave-length of 1.00001 Å at beamline 3W1A of the Beijing Synchrotron Radiation Facility. The dataset was indexed, integrated and scaled using the HKL2000 package^41^ and converted to amplitude using the CCP4 suite^42^. Data collection and processing results are summarized in Table S1.

The crystal structure of the complex was solved by molecular replacement using AutoMR^43^ implemented in Phenix^44^. The structures of unliganded HIV-1 clade A/E strain 93TH057 gp120 core (PDB code 3TGT) and the CD4 portion of the ternary complex of a HXBc2 gp120 core with CD4 and 17b (PDB code 1GC1) were used as search models. The initial model was further built manually using COOT^45^ and refined with Phenix. Simulated annealing, positional refinement and B-factor refinement were used in multiple rounds to improve the overall quality of the structure. Ordered water molecules were added to the structure in the last round of refinement. Refinement statistics are summarized in Table S1. All structural figures were prepared using Pymol (http://www.pymol.org/).

### HIV-1 Env-mediated cell–cell fusion assay

To determine the membrane fusion activity of Env from different clades and their mutants, cell-cell fusion was monitored using a reporter gene assay based on activation of a HIV-1 LTR-driven luciferase cassette in TZM-bl target cells by HIV-1 Tat from COS-1 effector cells, as described previously^24^. Briefly, COS-1 cells were transfected in 6-well plates with pSRHS-gp160 (1.0 μg/well). Cells transfected with pcDNA3.1 (1.0 μg/well) were used as a negative control. 24 h after transfection, cells were trypsinized (0.25% trypsin, HyClone), washed, and resuspended in fresh complete medium. To initiate cell-cell fusion, roughly equal numbers of TZM-bl cells were overlaid on confluent COS-1 cells in 12-well plates and co-incubated overnight. After co-incubation, cells were washed and lysed in reporter lysis buffer (100 μl/well), and assayed for luciferase activity according to manufacturer’s specifications (Promega, USA).

### Cell-associated pseudotyped HIV-1 infection assay

To further determine the membrane fusion activity of Env from different clades and their mutants, cell-associated pseudotyped HIV-1 infection assay was performed as described previously^46^. 293 T cells were co-transfected with backbone plasmid NLENY1-ES-IRES and HIV-1 envelope expression vector pSRHS-gp160 using Lipofectamine2000 (Invitrogen, USA). After 48 h, the culture supernatants were removed through centrifugation at 1500 g for 10 min. The remaining 293 T cells were trypsinized (0.25% trypsin, HyClone), washed and resuspended in fresh complete medium. To initiate cell-associated pseudotyped HIV-1 infection, roughly equal numbers of TZM-bl cells were overlaid on transfected 293 T cells in 12-well plates and co-incubated at 37 °C overnight. The remaining procedure was the same as cell–cell fusion test.

### CD4-associated pseudotyped HIV-1 infection assay

To determine the membrane fusion activity of CD4 mutants, CD4-associated pseudotyped HIV-1 infection assay was performed based on previously described methods^47^,^48^. HeLa cells expressing both CD4 and CCR5 were used as the target cells. To generate CD4 and CCR5 expressing HeLa cells, VSV-G pseudotyped viruses were packaged in 293 T cells by co-transfection of plasmids pMDLg/pRRE, pRev, pVSV-G and pQCXIP-CD4-IRES-CCR5. After 48 h, the culture supernatants were collected to infect HeLa cells for 24 h. To initiate CD4-associated pseudotyped HIV-1 infection, 293 T cells were co-transfected with backbone plasmids pEnv-CH70 and pNL4-3-Env(−)Luc(+) using Lipofectamine2000 (Invitrogen, USA) to produce pseudotyped HIV-1. After 48 h, the pseudotyped HIV-1 were collected through centrifugation at 1500 g for 10 min and were overlaid on CD4 and CCR5 expressing HeLa cells in 6-well plates at 37 °C for overnight. The remaining procedure was the same as cell–cell fusion test.

### Western blotting

Cells used above were analyzed by lysing transfected cells in NP-40 lysis buffer containing 1% PMSF for 30 min on ice. After centrifugation (12000 g, 15 min, 4 °C), the supernatant was separated by SDS-PAGE. Proteins were transferred to a 0.45 μm PVDF membrane (Millipore, USA) for 1 h at 100 V, 4 °C in a Bio-Rad Mini Transblot apparatus (Bio-Rad, USA). The membrane was blocked in 5% skim milk (in PBS buffer) for 50 min at RT (room temperature) to quench unspecific binding. Primary antibody was diluted at 1:1000 in blocking buffer and membranes were incubated for 90 min at RT. Membranes were washed 5 times for 5 min each. The appropriate horseradish peroxidase-conjugated secondary antibody was diluted at 1:6000 in blocking buffer and applied to the membrane for 45 min at RT. Protein bands were visualized on the Tanon-5200 enhanced chemiluminescent imaging system (Tanon, China).

### Alignment of gp120 and CD4 sequences

HIV gp120 and CD4 sequences from various strains or different species (http://www.hiv.lanl.gov) were aligned using CLUSTAL X program and drawn by WebLogo (http://weblogo.berkeley.edu/logo.cgi). Only regions associated with this study were shown.

### Molecular dynamics simulation

The missing chains in the gp120 were added by Modeller^49^. All disulphide and glycoside bonds resolved in the gp120-CD4 complex with a non-canonical interface were preserved by the tleap module of AMBER14^50^ with the FF14SB force field^51^. Then the complex was placed in a TIP3P^52^ water box. The distance between the edges of the box and the closest atoms of the complex was 10 Å. Cl- ions^53^ were added as counterions to neutralize the system.

2500 steps of steepest descent minimization and 2500 steps of conjugate gradient minimization were performed for the system, followed by a 500 ps constant volume MD simulation (NVT) to heat the system from 0 K to 300 K and a 500 ps constant pressure MD simulation (NPT, P = 1 atm). The Langevin thermostat^54^ was used for temperature control. During equilibration, a weak force constant of 10 kcal ∙ mol^−1^ ∙ Å^−2^ was applied on the complex as a harmonic constraint. Consequently, a 100-ns MD production was performed in the NPT ensemble at 300 K with a 2-fs step. As the complex was modeled to study the non-canonical interface, the harmonic constraint mentioned above was applied on the main chain of the complex in case of destabilization during the first 5 ns of the trajectory. The cut-off value of the Van der Waals interactions was set to 10 Å. The long-range electrostatic interaction was calculated by the Particle Mesh Ewald (PME) method^55^. The SHAKE algorithm^56^ was used to restrain all of the bond lengths that involved hydrogen atoms. Snapshots of the system were saved every 100 ps.

The CPPTRAJ module^57^ of AmberTools15 was used to calculate the root mean square deviations (RMSD) of the whole MD production, the average structure, and the occupancy of the hydrogen bonds of the last 10 ns trajectory. MMPBSA method^58^,^59^,^60^ was used to calculate the binding free energy between gp120 and CD4 of the last 10 ns MD simulation.

## Additional Information

**How to cite this article:** Duan, L.-W. *et al*. A non-canonical binding interface in the crystal structure of HIV-1 gp120 core in complex with CD4. *Sci. Rep.* **7**, 46733; doi: 10.1038/srep46733 (2017).

**Publisher's note:** Springer Nature remains neutral with regard to jurisdictional claims in published maps and institutional affiliations.

## Supplementary Material

Supplementary Information
