# Supplementary material for: A non-canonical binding interface in the crystal structure of HIV-1 gp120 core in complex with CD4
Source: Sci Rep. 2017 Apr 21;7:46733. doi: 10.1038/srep46733 (PMC5399459; doi:10.1038/srep46733)
Supplement: Supplementary Information [file srep46733-s1.pdf]

A non-canonical binding interface in the crystal structure of HIV-1 gp120 core in complex with CD4

Liang-Wei Duan[1,2‡], Hui Zhang[1‡], Meng-Ting Zhao[1], Ji-Xue Sun[3], Wen-Li Chen[1], Jian-Ping Lin[3] and Xin-Qi Liu[1*]

1 State Key Laboratory of Medicinal Chemical Biology, College of Life Sciences, Nankai University, Tianjin 300071, China

2 Research Center for Immunology, School of Laboratory Medicine, Henan Collaborative Innovation Center of Molecular Diagnosis and Laboratory Medicine, Xinxiang Medical University, Xinxiang 453000, China.

3 State Key Laboratory of Medicinal Chemical Biology, College of Pharmacy, Nankai University, Tianjin 300071, China

* Correspondence and requests for materials should be adressed to X.-Q.L. (liu2008@nankai.edu.cn)

‡ These authors contribute equally to this work.

Running title: Structure of a gp120-CD4 binary complex

Keywords: Crystal structure; HIV-1; gp120; conformational change; non-canonical binding interface; ibalizumab

Table S1. Data collection and refinement statistics.

**Data collection**	
Crystal	Gp120 core$_{V3e}$ and CD4$_{D1D2}$ complex
Space group	$P2_1$
Wavelenth (Å)	1.00001
Cell constants (Å/ °)	a = 68.444, b = 66.468, c = 73.850
	α = 90.0, β = 108.4, γ = 90.0
Molecules per ASU$^{\Phi}$	1
Resolution (Å)*	50-2.47 (2.49-2.47)
Completeness (%)*	95.6 (96.8)
Redundancy*	3.0 (2.7)
No. of total reflections	66115
No. of unique reflections	21690
I/σ (I)*	21.6 (2.15)
R_{sym} (%)*†	4.9 (47.4)
**Refinement**	
Resolution (Å)	50-2.47
No. of reflections	21650
R_{work}/R_{free} (%)‡§	19.83/25.67
No. of atoms	
Protein	4148
Water	140
B-factors (Å^2)	
Protein	40.11
Water	40.29
R.m.s. deviations	
Bond length (Å)	0.009
Bond angle (°)	1.288
Ramachandran analysis	
Favored (%)	94.3
Allowed (%)	5.7
Outliers (%)	0

* Highest resolution shell is shown in parentheses.

$^{\Phi}$ ASU=asymmetric unit.

† $R_{sym} = \Sigma|I-<I>|/\Sigma<I>$, where I is the observed intensity, and <I> is the average intensity of multiple observations of symmetry related reflections.

‡ R = Σhkl||Fobs|-|Fcalc||/Σhkl|Fobs|

§ R_{free} is calculated from 5% of the reflections excluded from refinement.

Table S2. The comparative analyses of canonical interface and non-canonical interface.

	Monomer	Nat[a]	Nres[b]	Monomer	Nat	Nres	Interface area($Å^2$)
Canonical interface	Gp120 core$_{V3e}$	95	24	CD4$_{D1D2}$	106	34	940
Non-canonical interface	Gp120 core$_{V3e}$	51	20	CD4$_{D1D2}$	53	15	471

[a]Nat represents the number of atoms in the relative interface.

[b]Nres represents the number of amino acid residue in the relative interface.

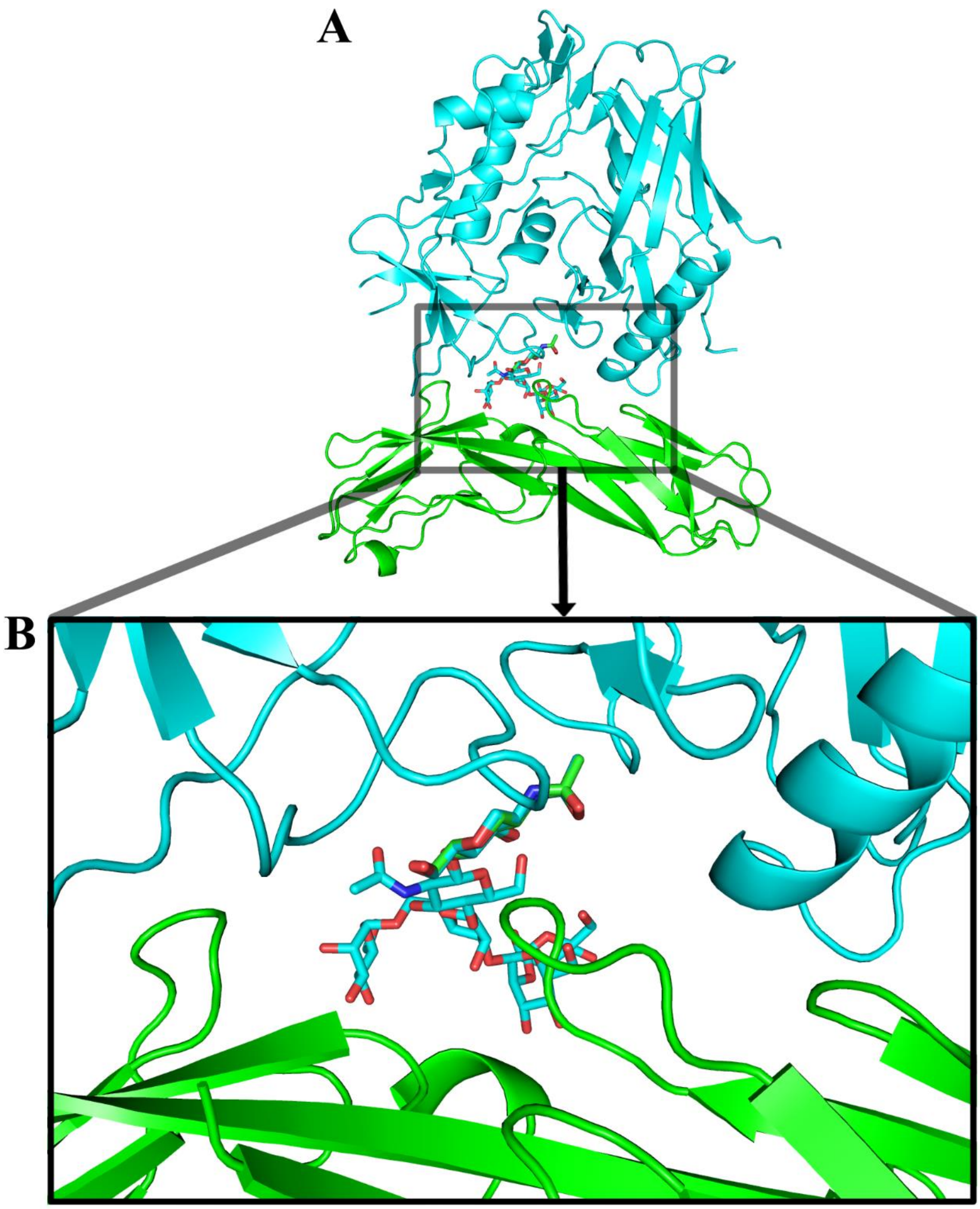

Figure S1. The localization of a long N-linked glycan (6 residues) in the non-

canonical interface. (**A**) Full view of the position of a long N-linked glycan in the non-canonical interface. (**B**) Zooming-in of the non-canonical interface. The first glycan NAG of the long N-linked glycan chain was shown as sticks colored according to atomic types: green for carbon, blue for nitrogen and red for oxygen. The other glycans of the long N-linked glycan were shown as sticks and colored according to atomic types: cyan for carbon, blue for nitrogen and red for oxygen. Gp120 was shown in cyan and CD4 was shown in green in these two pictures.

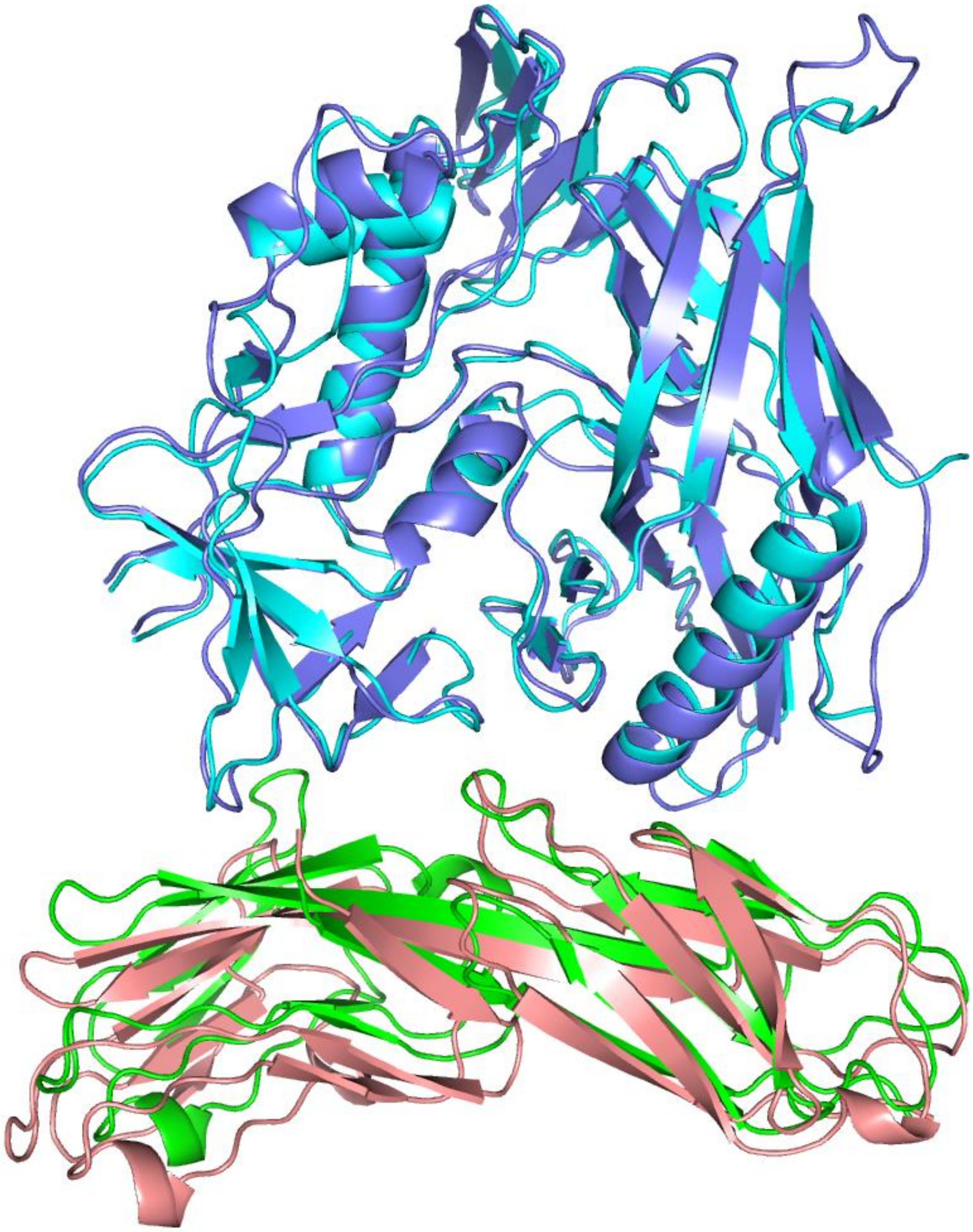

Figure S2. Comparison of CRF07_BC gp120 core$_{V3e}$-CD4$_{D1D2}$ complex with

simulative structure obtained through molecular dynamics simulations. **G**p120 and CD4 of CRF07_BC gp120 core$_{V3e}$-CD4$_{D1D2}$ complex were shown in cyan and green respectively. Gp120 and CD4 of simulative structure were shown in slate and salmon respectively.

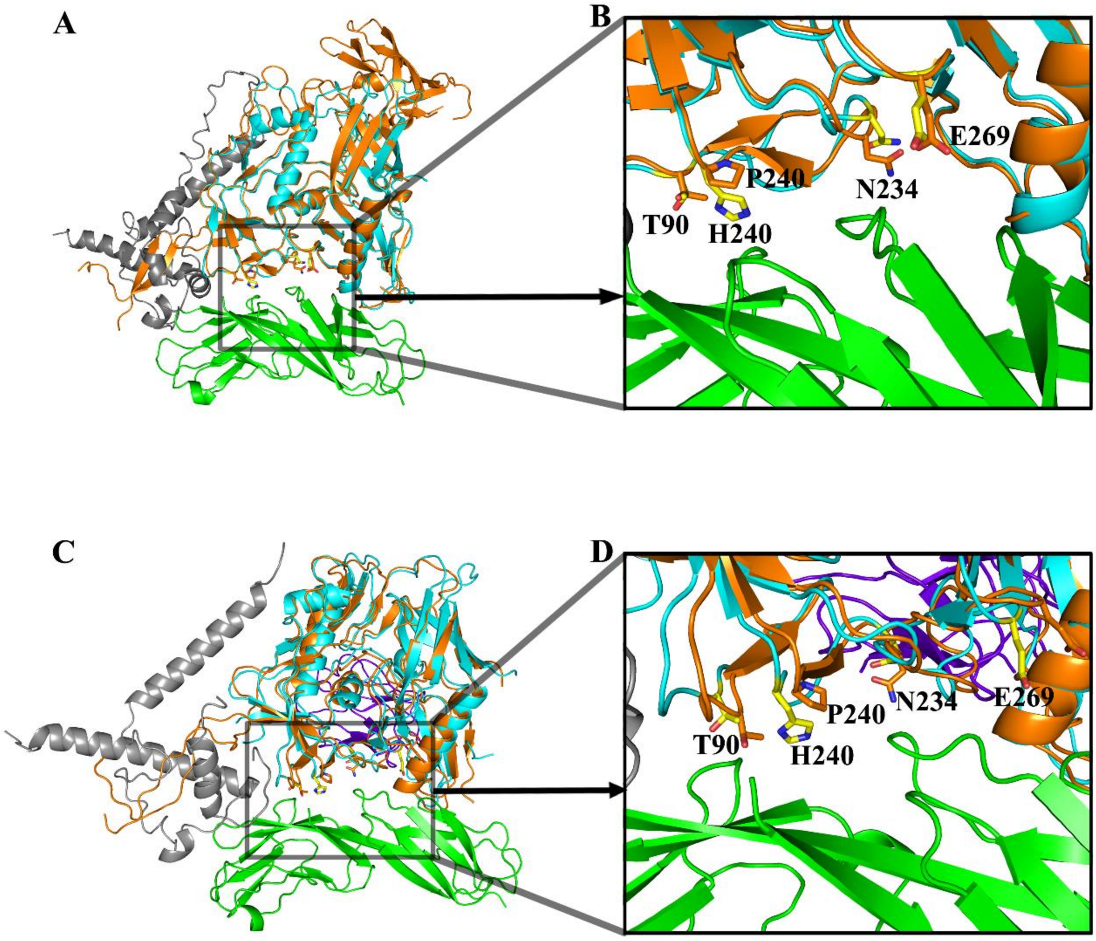

Figure S3. Comparison of the conformation of the identified gp120 residues in CRF07_BC gp120 core$_{V3e}$-CD4$_{D1D2}$ complex with pre-fusion BG505 SOSIP.664 gp140 with closed state (PDB code 5CEZ) and BG505 SOSIP.664 Env-sCD4 complex with open state (PDB code 5THR). (A) The identified gp120 residues in CRF07_BC gp120 core$_{V3e}$-CD4$_{D1D2}$ complex and in pre-fusion engineered BG505 SOSIP.664 gp140. Two complexes were superimposed based on gp120 region. The residues in two complexes were marked in a rectangle. Gp120 and CD4 of CRF07_BC gp120 core$_{V3e}$-CD4$_{D1D2}$ complex were shown in cyan and green respectively. Gp120 and gp41 of pre-fusion engineered BG505 SOSIP.664 gp140 were shown in orange and gray respectively. (**B**) Zooming-in of the non-canonical interface. Side chains of the identified gp120 residues in CRF07_BC gp120 core$_{V3e}$-CD4$_{D1D2}$ complex were shown as sticks and colored according to atomic types: yellow for carbon, blue for nitrogen and red for oxygen. Side chains of the identified gp120 residues in pre-fusion engineered BG505 SOSIP.664 gp140 were shown as sticks and colored according to atomic types: orange for carbon, blue for nitrogen and red for oxygen. (**C**) The

identified gp120 residues in CRF07_BC gp120 core$_{V3e}$-CD4$_{D1D2}$ complex and in BG505 SOSIP.664 Env-sCD4 complex. Two complexes were superimposed based on gp120 region. The residues in two complexes were marked in a rectangle. Gp120, gp41 and CD4 of BG505 SOSIP.664 Env-sCD4-17b-8ANC195 complex were shown in orange, gray and purpleblue respectively. **(D)** Zooming-in of the non-canonical interface.

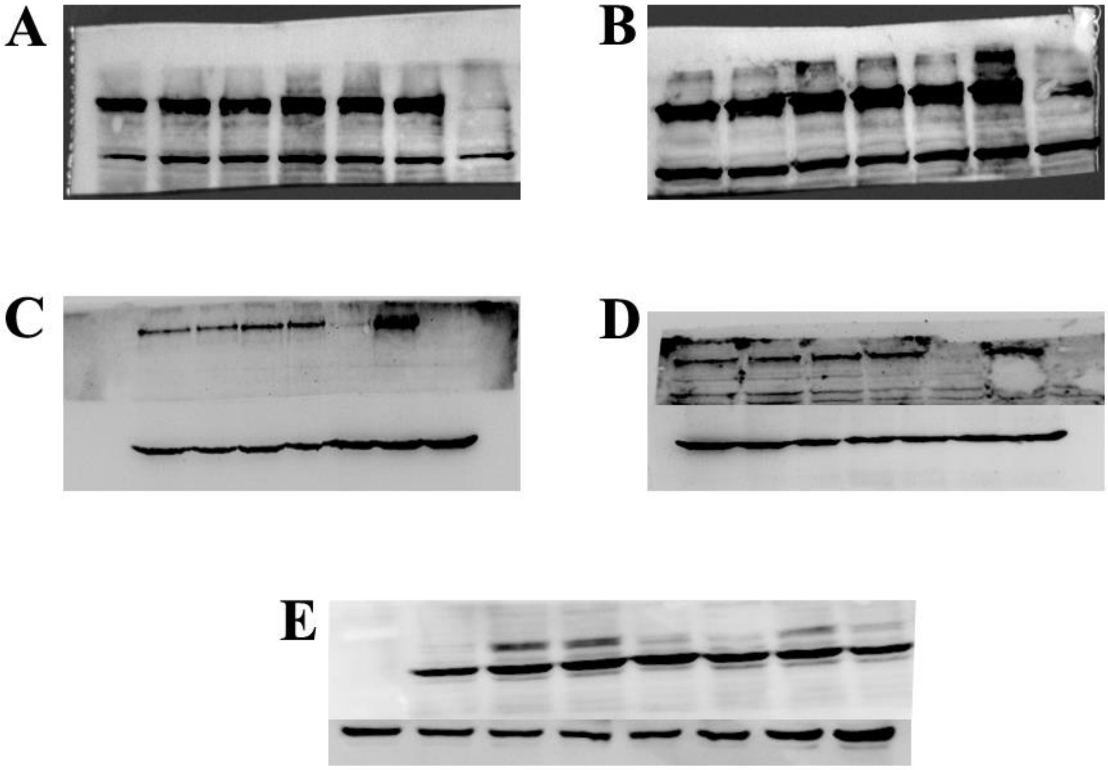

Figure S4. The full-length gels/blots which were used to generate the cropped gels/blots in the manuscript. (A) The gel/blot in Figure 3A. **(B)** The gel/blot in Figure 3B. **(C)** The gel/blot in Figure 3C. **(D)** The gel/blot in Figure 3D. **(E)** The gel/blot in Figure 4B.
